# Exploring the effectiveness of dextroamphetamine for the treatment of stimulant use disorder: a qualitative study with patients receiving injectable opioid agonist treatment

**DOI:** 10.1186/s13011-021-00399-2

**Published:** 2021-09-16

**Authors:** Heather Palis, Kirsten Marchand, Gerald “ Spike” Peachey, Jordan Westfall, Kurt Lock, Scott MacDonald, Jennifer Jun, Anna Bojanczyk-Shibata, Scott Harrison, David C. Marsh, Martin T. Schechter, Eugenia Oviedo-Joekes

**Affiliations:** 1 Centre for Health Evaluation & Outcome Sciences, Providence Health Care, St. Paul’s Hospital, 575- 1081 Burrard St, Vancouver, BC V6Z 1Y6 Canada; 2 Anti-Stigma Zone, Vancouver, Canada; 3 Canadian Association for Safe Supply, Vancouver, Canada; 4 Providence Health Care, Providence Crosstown Clinic, 84 West Hastings Street, Vancouver, BC V6B 1G6 Canada; 5 Northern Ontario School of Medicine, 935 Ramsey Lake Road, Sudbury, ON P3E 2C6 Canada; 6 Canadian Addiction Treatment Centres, 175 Commerce Valley West, Suite 300, Markham, Ontario L3T 7P6 Canada; 7 ICES North, 41 Ramsey Lake Rd, Sudbury, ON P3E 5J1 Canada; 8 Health Sciences North Research Institute, 56 Walford Rd, Sudbury, ON P3E 2H2 Canada; 9 School of Population and Public Health, University of British Columbia, 2206 East Mall, Vancouver, BC V6T 1Z3 Canada

**Keywords:** Stimulant use disorder, Cocaine use, Methamphetamine use, Dextroamphetamine, Injectable opioid agonist treatment

## Abstract

**Background:**

A high proportion of people receiving both oral and injectable opioid agonist treatment report concurrent use of stimulants (i.e. cocaine and or amphetamines), which has been associated with higher rates of continued illicit opioid use and treatment dropout. A recent randomized controlled trial demonstrated the effectiveness of dextroamphetamine (a prescribed stimulant) at reducing craving for and use of cocaine among patients receiving injectable opioid agonist treatment. Following this evidence, dextroamphetamine has been prescribed to patients with stimulant use disorder at a clinic in Vancouver. This study investigates perceptions of the effectiveness of dextroamphetamine from the perspective of these patients.

**Methods:**

Data were collected using small focus groups and one-on-one interviews with patients who were currently or formerly receiving dextroamphetamine (*n* = 20). Thematic analysis was conducted using an iterative approach, moving between data collection and analysis to search for patterns in the data across transcripts. This process led to the defining and naming of three central themes responding to the research question.

**Results:**

Participants reported a range of stimulant use types, including cocaine (*n* = 8), methamphetamine (n = 8), or both (*n* = 4). Three central themes were identified as relating to participants’ perceptions of the effectiveness of the medication: 1) achieving a substitution effect (i.e. extent to which dextroamphetamine provided a substitution for the effect they received from use of illicit stimulants); 2) Reaching a preferred dose (i.e. speed of titration and effect of the dose received); and 3) Ease of medication access (i.e. preference for take home doses (i.e. carries) vs. medication integrated into care at the clinic).

**Conclusion:**

In the context of continued investigation of pharmacological treatments for stimulant use disorder, the present study has highlighted how the study of clinical outcomes could be extended to account for factors that contribute to perceptions of effectiveness from the perspective of patients. In practice, elements of treatment delivery (e.g. dosing and dispensation protocols) can be adjusted to allow for various scenarios (e.g. on site vs. take home dosing) by which dextroamphetamine and other pharmacological stimulants could be implemented to provide “effective” treatment for people with a wide range of treatment goals and needs.

## Background

Injectable opioid agonist treatment (iOAT) with either hydromorphone (an opioid analgesic) or diacetylmorphine (pharmaceutical-grade heroin) is an effective treatment for opioid use disorder among people not benefitting from oral opioid agonist treatments (OAT) [1, 2]. A high proportion of people receiving both oral and injectable OAT have been found to report concurrent use of stimulants (i.e. cocaine and or amphetamines) [3–5]. While OAT has not been associated with increases in illicit stimulant use, reductions overall remain modest and the ongoing use of illicit stimulants in OAT has been associated with higher rates of continued illicit opioid use and treatment dropout [6, 7].

While a number of clinical trials have investigated various pharmacological treatments for stimulant use disorder, many of these trials have had small sample sizes and high treatment discontinuation rates [8, 9]. Therefore, there has been a paucity of evidence derived from clinical trials regarding the overall effectiveness of pharmacological treatments for stimulant use disorder. Nevertheless, the Cochrane Collaboration’s review of psychostimulant drugs for the treatment of cocaine use disorder concluded that promising although modest results existed for dextroamphetamine (a pharmacological stimulant) [8]. As such, in 2016, a double-blind randomized controlled trial was conducted in the Netherlands to investigate the effectiveness of dextroamphetamine for the treatment of cocaine use disorder among iOAT patients [10]. Based on the primary outcome of abstinence (i.e. no use of illicit stimulants), the authors concluded effectiveness of the medication compared to placebo. The authors also considered secondary outcomes, including cravings, days of cocaine use (to measure reductions in use where abstinence was not achieved), and health and psychosocial outcomes [11].

The transition toward incorporating a diverse range of outcome measures in the study of treatments for stimulant use disorder has been acknowledged in recent literature in the field. For example, a systematic review concluded that abstinence as a primary study endpoint may not reflect patient treatment goals for patients with stimulant use disorder and therefore does not reflect a sensitive marker of clinically meaningful change in substance use [12]. Furthermore, a recent meta-analysis of prescription psychostimulants for the treatment of stimulant use disorder found these medications to be efficacious for the treatment of cocaine use disorder when provided in higher doses [13].

Following the evidence from the Netherlands, dextroamphetamine prescribing was integrated into standard of care for patients with stimulant use disorder in an iOAT clinic in Vancouver (Canada) in 2018. This is the first time to our knowledge that dextroamphetamine has been offered to iOAT patients outside of clinical trial, and represents the first clinical setting in North America to routinely offer dextroamphetamine as a treatment for stimulant use disorder alongside treatment for opioid use disorder. Given the modest effects of prescribed stimulants to date, and continued study of pharmacological stimulants, it is important to consider the nuances of how the medication works for each patient. Since there is little empirical and clinical literature on dextroamphetamine in the context of opioid use disorder, there is still much to know about how the medication works and an important first step is to discuss treatment experiences with patients.

Dextroamphetamine prescribing in this iOAT clinic offers a unique opportunity to capture patient perceptions of the medication. The study inquired about patients’ experiences receiving dextroamphetamine for the treatment of stimulant use disorder, accounting for their views of both the medication itself and the way it was delivered (process of delivery) with the intention of understanding how these experiences impacted on their perceptions of its effectiveness. The central research question investigated was: “What experiences influence patients’ perceptions of the effectiveness of dextroamphetamine for the treatment of stimulant use disorder?”

## Methods

### Sample and setting

Participants of the present study had formerly taken part in the Research on the Utilization of Therapeutic Hydromorphone (RUTH) (2016–2019) study which investigated the treatment experiences and outcomes of patients receiving iOAT at a community clinic in Vancouver [14]. Participants visited this clinic daily to receive iOAT for the treatment of opioid use disorder, and beginning in 2018 patients who reported cocaine and/or methamphetamine use were offered and prescribed (if desired) dextroamphetamine for the treatment of stimulant use disorder. RUTH participants who consented to being contacted for future studies and who reported illicit stimulant use (i.e. crack cocaine, cocaine powder or methamphetamine) were eligible to take part in the study. Participants were recruited by their preferred method of contact as reported in the RUTH study (either by telephone or in person).

### Data collection

Focus groups and one-on-one interviews were conducted between December 2019–December 2020. Given little existing data on the subject matter, data collection began with small focus groups (*n* = 3 groups with 8 participants total) to explore participants’ experiences receiving the medication. Discussions were semi-structured and focused on the following subject areas: a) benefits of the medication itself; b) challenges of the medication itself; c) benefits of the process of medication delivery; d) challenges of the process of medication delivery. In the focus groups, questions remained broad and open-ended to allow the participants to guide the discussion. Examples of the broad questions that were posed of participants include: *“What have been the benefits you have experienced from taking this medication?; What have been the challenges you have experienced from taking this medication?; What sorts of positive outcomes have you experienced?; What sorts of negative outcomes have you experienced?”; “What about the process of receiving dextroamphetamine at Crosstown Clinic have you liked?”; “What could be done to improve the process of receiving dextroamphetamine at Crosstown Clinic?”*. Questioning became more focused within each of these topic areas in the subsequent one-on-one interviews.

Focus group participants were sampled to represent a range of illicit stimulant use profiles (i.e. cocaine vs. crystal methamphetamine, injection vs. smoked) and engagement with dextroamphetamine (current vs. former) to capture perceptions from people with a diversity of experiences receiving dextroamphetamine [15]. Following the focus groups, one-on-one interviews were conducted with participants who had not taken part in the focus groups. Additional sub-questions relating to specifics of the medication and the process of medication delivery were integrated into the topic guide to be posed in the one-on-one interviews. For example, focus group participants outlined wanting to receive take home doses so in subsequent interviews we asked participants: *“Do you want to receive take home doses? If so, why? What benefit would this offer that you do not have now?”* Interview participants were also asked about their motivations for street stimulant use as this was a topic area raised by focus group participants as related to their experience with dextroamphetamine. Interviews continued (*n* = 12 interviews conducted one-on-one) until all eligible participants who could be reached were included in the final sample (*n* = 20).

Data collection began in-person at a confidential research office that was familiar to participants and in close proximity to the iOAT clinic, including all 3 focus groups, and the first 4 interviews. Due to COVID-19 physical distancing protocols, after March 2020, all remaining data collection (*n* = 8 interviews) was conducted by telephone. Given the interviewer’s pre-existing rapport with participants, the transition to telephone interviews did not seem to impact the quality of the information collected (i.e. flow of conversation, coverage of questions). Focus groups lasted on average 45 min, and interviews had an average duration of 25 min. Both focus groups and interviews were audio recorded. Recordings were transcribed verbatim and, identifying details were removed, and pseudonyms were assigned to each participant. All participants were compensated $30. Ethical approval was provided by the University of British Columbia/Providence Health Care Behavioural Research Ethics Board (H19–02514) and participants provided written informed consent prior to beginning data collection.

### Data analysis

Thematic analysis [16] was selected given the goal of identifying, analysing and reporting on patterns (themes) in the data across participants who had received dextroamphetamine. Prior to conducting analyses, transcripts were reviewed to gain familiarity with the content. Analysis began with an inductive, data-driven approach, whereby codes were attached to the data to directly reflect participants’ words, rather than applying a pre-existing coding framework. Coding was conducted by author HP who has extensive experience collecting and analyzing qualitative research data with patients in the iOAT setting [17–19]. Data collection began with broad questioning about the benefits and challenges of the medication, and subsequent data collection included more specific and focused questioning on sub-topics identified as important in initial data collection. This was accomplished using an iterative approach, whereby initial codes generated from analysis of the focus group data offered insights into important areas of further inquiry for subsequent interviews. Team members met throughout the stages of analysis to discuss the coded content and to inform next steps in data collection. For example, in the focus groups participants discussed their motivations for street stimulant use as related to their experiences receiving dextroamphetamine. After consultation between team members (HP, EOJ, GP, DSM), the decision was made to incorporate questioning about participants’ motivations for street stimulant use into following interviews.

The approach of constant comparison was applied throughout the stages of coding, comparing new data to existing data, and creating new codes where data did not fit existing codes. Initial codes were raised to themes by searching for patterns in the data across all transcripts and by reviewing analytic memos where reflections on these patterns were documented. This process led to the defining and naming of three central themes responding to the research question. Analysis of all collected data (focus groups and interviews) was conducted in one project in NVivo 12 [20].

## Results

### Participants’ stimulant use profiles

Participants were sampled from the RUTH study, which represented a cohort of people who had reported on average over 15 years of illicit stimulant use (i.e. cocaine and/or crystal methamphetamine) at baseline prior to initiating iOAT [5]. Table 1 displays the demographic and stimulant use profiles of participants of the qualitative interviews, and of the RUTH study. Following the sampling strategy, a diversity of stimulant use profiles are represented. The sample also had a broad range of self-reported exposure to the medication (reported to range from approximately 1 week to approximately 3 years total), and included people currently (*n* = 7) and formerly (*n* = 13) receiving dextroamphetamine. Of those who had discontinued dextroamphetamine, some had an interest in receiving it again in the future, while others did not. Five of these 13 participants had transitioned to receiving another prescribed stimulant (methylphenidate (Ritalin)) which was offered for patients not responding to dextroamphetamine.

**Table 1:** Demographic and stimulant use profiles of study participants (*n* = 20) and RUTH study sample (*N* = 131)

	N = 20 N (%)	N = 131 N (%)
**Gender**		
Man	17 (85.0)	94 (71.7)
Woman	3 (15.0)	37 (28.2)
Average age (years)	53.65 ± 7.84	48.63 ± 9.31^a^
**Illicit stimulant use profiles**^**b,c**^		
**Any Cocaine use**	12 (60.0)	50 (38.2)
Crack cocaine smoked	5 (41.6)	45 (90.0)
Cocaine powder injected	7 (58.3)	21 (42.0)
**Crystal methamphetamine use**	12 (60.0)	41 (31.3)
Crystal methamphetamine smoked	1 (8.3)	1 (2.4)
Crystal methamphetamine injected	11 (91.7)	40 (97.6)

^a^RUTH participants’ ages were self-reported at baseline in 2016.

^b^For qualitaivte interview participants stimulant use profiles reflect those self-reported at time of dextroamphetamine initiation. For RUTH participants, stimulant use profiles reflect those self-reported at the RUTH study’s baseline assessment.

^c^Four participants reported injection of both cocaine and methamphetamine

### Findings of thematic analysis

There were three central themes identified as related to participants’ experiences receiving dextroamphetamine which influenced their perceptions of the effectiveness of dextroamphetamine as a treatment for stimulant use disorder. These were**:** (1) achieving a substitution effect; (2) reaching an adequate dose; and (3) ease of medication access.

#### Achieving a substitution effect

Participants sought a range of effects from the use of illicit stimulants. Perceptions of the effectiveness of dextroamphetamine as a treatment for stimulant use disorder were impacted by the extent to which it was seen to provide a substitution for the effect they received from the illicit stimulant they were using. For example, one participant who had previously attempted dextroamphetamine but was no longer receiving it described what he felt the medication would have to do for him to be able to stop using illicit stimulants:

*“If it [Dextroamphetamine] is going to be a maintenance drug it would have to have the ability to take the place of the drug you are trying to get people to stop using.”- Jason, 40 year old man*

The most commonly sought effects from dextroamphetamine was a boost of energy, alertness, or focus. Among many participants for whom the intended effect was a boost of energy, dextroamphetamine was reported to offer this effect. For example, one participant who reported injecting methamphetamine daily to help with wakefulness described that he gained this effect from dextroamphetamine:

*“I wanted to get off of jib [crystal methamphetamine], or at least slow down. I have sleep apnea really bad and I need to stay awake and alert, that’s why I was taking jib, just to stay awake. I used Dextroamphetamine instead. It did what I wanted it to do, it kept me awake when I wanted to be awake, for me it is like a win win situation.” – William, 62-year-old man*

For some participants, the desired energy was sought to support them in completing tasks and gaining focus that they otherwise struggled to find in the absence of the medication. This was discussed in the context of self-reported attention deficit hyperactivity disorder (ADHD) diagnosis by a few participants, who described feeling that dextroamphetamine helped to “*slow things down*” or to provide “*focus*” (*Jennifer- 43-year-old woman*). One participant who had been regularly using cocaine and methamphetamine for many years described:

“*It helps me to keep my thoughts focused and not scrambled, I am able to retain information, clean the house, just doing chores, you are a little more focused and you feel like you know what has got to be done and you just do it. [Before] I would not have had any interest in doing that, the difference now is it [dextroamphetamine] gives me what I need to speed up, to wake up and get with the program. The dexy [dextroamphetamine] seems to help me pull it all together. It kind of picks you up to a normal pace I don’t feel like I am going to fall asleep I don’t feel like I am gonna be wide awake I just feel like I am ok…I am not like lost in the fog anymore, I pulled out of the fog. I am awake.”- Dean, 59-year-old man*

For some participants, dextroamphetamine was sought to support them in managing cravings for illicit stimulants. This was particularly true for people who were looking to fully stop their illicit stimulant use. For some participants, cravings were managed to the extent that they no longer had an interest in the feeling provided by street stimulant use. For example, one participant who had been smoking crack cocaine daily described that the “taste” or craving for use was stopped by dextroamphetamine:

*“It [dextroamphetamine] curbed the thriving for it [crack cocaine], you know. I was using it just to cut down on craving. Doing crack [cocaine] while you are on it doesn’t feel right it just turns me right off it. Dextroamphetamine kills the taste for it [crack cocaine]...I just want to get off it [crack cocaine]. All my money went to it- everything that I made went into it, I worked for it, I gave everything to it, and now I just want off it.” – Elijah, 52-year-old man*

For participants for whom the intended effect of their stimulants was to *get a “rush” or feeling of “euphoria”*, dextroamphetamine did not provide a substitution effect. This was often discussed by referencing the preference for a short acting stimulant, where dextroamphetamine, because of its long-acting nature, was not seen as a “*viable substitute*” (*Jason- 40-year-old man*). For example, one participant who injected cocaine daily reported that the effect he gained from dextroamphetamine was not comparable to that of cocaine:

*“It [cocaine] is like almost a rush of energy running through my body right. It is very euphoric and very addictive. My biggest problem with getting amphetamines for replacement is that they have too much of an afterlife right. I like cocaine because it’s short lived. I am high as a kite and I am down and normal in an hour, you wouldn’t even know I did it an hour later, but speed [dextroamphetamine] and all the other substitutes have a long half-life and it takes a couple hours to hit you and then it lasts another half evening and then you still feel the effects the next day.” -Leonard, 51-year-old man*

#### Reaching a preferred dose

Perceptions of the effectiveness of dextroamphetamine as a treatment for stimulant use disorder were tied closely to patients’ dose preferences. The speed with which the dose was increased (titration) and the maximum dose received were both important contributors to dose preferences. For some participants, these two things were closely connected, where they did not reach a dose that was high enough from their perspective, because the starting dose was not enough to attract them to the medication or to lead them to believe that it could possibly have any effect for them. Upon reflection (particularly in focus groups where people heard from their peers) some participants reconsidered and reported wanting to try dextroamphetamine again at a higher dose or give it a chance for a longer period of time.

The dosing protocol at Crosstown Clinic involved prescribed daily doses up to 120 mg, 60 mg dispensed twice daily. Many participants made comparisons between the potential effect of this dose as compared to the quantity of methamphetamine or cocaine they currently used. Some participants viewed the maximum dose of 120 mg as inadequate. In this context, sometimes the desire for a higher dose was discussed, while for others the preference for an instant release formulation medication was discussed. For example, one participant who reached the 120 mg daily dose reported that he still did not achieve the effect he was seeking, and did not see dextroamphetamine as a suitable substitute:

*“I want like a powerful stimulant that I guess keeps me awake and energized and makes me do my weird repetitive behaviors. I couldn’t picture going and asking the doctor to give me from like 10 pills to 35 dextroamphetamine pills or something. That is what the dose would need to be to maybe be [what I need]. It would obviously come to a point where it would have an effect but I don’t want to be like having to take a ridiculous amount of pills. I wish Adderall had been covered so I could have been given a chance to try that just to see, I have a feeling that might have been a lot more effective” – Jason, 40-year-old man*

It is important to note that communication with the prescriber when beginning the prescription was important to reaching an effective dose. This was challenging for patients who reported not regularly engaging in iOAT, or for people who were managing other concerns related to their health. For example, one participant who injected crystal methamphetamine multiple times daily described not having regular check-ins with his prescriber and stopping the medication prior to reaching an effective dose:

*“I should have told them [prescriber] that it wasn’t enough but I went long periods without seeing them…I never actually approached them and said I want you to up the dose…I think it could be a positive thing If you get enough, for other people I have heard lots of good things. I would try it again because I would like to get off speed [crystal methamphetamine].” -Jeremy, 51-year-old man*

Participants also discussed the speed of the titration protocol and suggested regular check-ins during the first few days on the medication to help ensure there would not be a preference to return to street stimulant use. Participants recommended starting at a higher dose (e.g. 30 mg twice per day rather than 15 mg twice per day), one that was “*enough to make a difference- to prevent them [the patient] from running out of the place and going to buy some dope”* (Michael- 66-year-old man) and titrating up faster to ensure the patient did not become *“disinterested”.* One participant who was currently receiving dextroamphetamine reported that he only gained benefit from the medication when he reached the maximum dose of 60 mg twice per day:

*“For a while there I was thinking I might as well just quit it [Dextroamphetamine]. I started at 15mgs twice a day… that was too low of a dose, I didn’t know it was gonna really work you know like I knew I was cutting back a little bit but I couldn’t see it as a means to an end. I went and started off with 2, and then went 2 more. When I got on 3 two times a day that started to help quite a bit, I didn’t need the jib [crystal methamphetamine], and it didn’t interfere with my sleep. Now I get 4 twice a day and it is enough to keep me going and it is not interfering with my sleep at all so it is working for me, but I think people should start off on a better dose.” -Aaron, 71-year-old man*

#### Ease of medication access

Overall, experiences relating to the ease with which the medication could be accessed were described to influence perceptions of the medication. Participants had diverse goals and different preferences for the level of engagement they had with their OAT and stimulant treatment. Some participants were content with the integrated model of delivery, where the medication was offered within the iOAT clinic. Participants saw this as a convenient form of medication delivery and appreciated the medication reminders and dispensation from the on site pharmacy. One participant who had been visiting the clinic daily for many years outlined the ease of access from her prescriber, and the convenience of having access to dextroamphetamine at the same location and at the same time as her other medication:

*“I liked the convenience of it. The fact that you could get it when you get your other meds. Like I didn’t have to go somewhere separate for it. It wasn’t very difficult for me to get it from my doctor.” – Elizabeth, 49-year-old woman*

Other participants reported the reliability and consistency of access to the medication, for example having the option to take the medication on days when they wanted and to leave it when they did not. Participants reported comfort in knowing that taking a break from the medication for a period of time would not hurt the possibility of receiving it in the future. For example, one participant who had been smoking crack cocaine for many years reported changing events in his life, deciding to return to using crack cocaine stating that the cocaine was the *“only thing in my life keeping me sane”.* He reported that he would like to try dextroamphetamine again in the future:

*“I stopped taking it a while ago, I was taking it but at this point in my life I am not trying to curb my crack intake...I probably will try it again… I have an open script with it so anytime I want to start it up again I just go and talk to the doctor and he will start writing it up again. So that has been nice, so anytime I want it it’s there, and knowing that it’s there is a big plus.”- Matthew, 55-year-old man*

Many participants described wanting to have access to take home dose (i.e. carries) of dextroamphetamine. This was seen to impact on the potential effectiveness of the medication, given for many people the timing of their craving for illicit stimulants did not line up with the timing with which they were able to receive the medication at the clinic, for example one participant shared *“my crack ridges [cravings] don’t follow any schedule”* (Matthew, 55 year-old man). Furthermore, participants engaged in different activities outside of the clinic each day, and for many, further flexibility in the delivery of this medication would be required in order to allow dextroamphetamine to fit into those routines. For example, one participant outlined that he would only see benefit from the medication when he had control over when he took it, and taking it at the same time as his iOAT dose was not always preferred:

*“If they give them [dextroamphetamine pills] to me [to take home] I would take them when I need them ... they have certain times they hand them out and I don’t need them right then. Sometimes when I need them, I don’t have anything to get me to where I want to be and I have to wait until the next time, whereas if I was given them I could take them when I need them… Sometimes at night I could use more, not always, but if I am doing something, I might want to have some at night.” – Dean, 59-year-old man*

Furthermore, one participant described that each day was a little different for him, where he might want to change the timing of dose administration depending on his work and sleep schedule. He also shared that medication diversion was not something he would consider and should not serve as a barrier to carries:

*“It [dextroamphetamine] works better if you can take it when you need it. Sometimes I am tired and can’t stay up it is nice to have it so I can keep it on me for when I need it. It keeps me awake for work right, and I work 3 days on and 3 off. And that’s what the carry does too, on my days off maybe I want to take it a little bit later if I want to have a nap and take it when I wake up. You know there is no reason why I could not take it for a month and quite easily dose myself. It’s not something I would sell; I work to make money and it’s just for my personal use. My body knows when I need it without having to walk down to the same place [iOAT clinic] every day just to get it.” – William, 62-year-old man*

## Discussion

In this study, three central themes were identified as influencing participants’ perceptions of the effectiveness of dextroamphetamine as a treatment for stimulant use disorder. These themes extend beyond traditional operationalizations of effectiveness in the study of pharmacological treatment for stimulant use disorder, which have often been focused on the pharmacological action of the medication and its impact on achieving abstinence [8, 9], and in some cases extend to consideration of secondary outcomes including reduced craving, and improved health and psychosocial outcomes [11]. In the present study, motivations for illicit stimulant use were diverse. Perceptions of dextroamphetamine’s effectiveness were often influenced by the extent to which this medication could provide a substitute for the effects offered by illicit stimulant use. This substitution effect often served as a pre-condition for supporting reduced illicit stimulant use, or abstinence in cases where this was the participant’s goal.

Participants who reported using illicit stimulants to gain a boost of energy in many cases reported that dextroamphetamine provided a substitute for these effects. This was true in the context of concurrent diagnosis of ADHD, where a few participants reported achieving improved alertness, and focus from dextroamphetamine. This is consistent with prior clinical trials, where dextroamphetamine has been shown to reduce cravings and use of cocaine, and to improve ADHD symptoms when prescribed to people with concurrent ADHD and cocaine use disorder [21]. Given the high rates of ADHD among people with stimulant use disorder [22, 23], and the indication of dextroamphetamine for the treatment of ADHD, ADHD screening could be incorporated into assessments for patients seeking treatment for stimulant use disorder with potential for dual benefits.

Beyond the desire for a boost of energy, many participants reported presenting to treatment with the goal of managing their cravings for illicit stimulant use. For some participants, dextroamphetamine was seen to be effective at reducing craving. This finding has been reflected in prior clinical trials, where subjective craving scores have been reduced among people receiving dextroamphetamine (compared to placebo) for both cocaine [10, 24] and amphetamine use disorder [25]. Lastly, some patients reported the euphoric effect or “rush” as their motivation for illicit stimulant use. In the present study, patients received extended-release dextroamphetamine. Extended-release stimulants are less likely to produce subjective effects compared to short-acting or instant release prescription stimulants, and can also decrease the euphoriant and reinforcing effects of (illicit) stimulant use due to cross-tolerance [26]. As such, for patients for whom the goal of illicit stimulant use was to achieve a euphoric effect, dextroamphetamine did not provide an effective “substitute”. As with treatment of other substance use disorders, not everyone will be engaged by currently available (while limited) treatments, and some will continue to use illicit stimulants. Nevertheless, in the absence of availability of alternative medications, the potential benefit of dextroamphetamine remains among people who continue to use illicit stimulants to achieve euphoric effects. For example, dextroamphetamine could support patients to reduce the frequency of illicit stimulant use [10, 11].

Another important contributor to perceptions of the effectiveness of dextroamphetamine in the present study was the dose received. Participants all received dextroamphetamine following a standard dosing protocol, beginning at 15 mg twice per day, and titrating up as tolerated. For many patients there was no perceived effect until a dose of up 90 or 120 mg was reached. Communication with the prescriber in this process was found to be important to remaining engaged with the treatment. This finding is consistent with what is known from prior studies on the delivery of other treatments for substance use disorder such as opioid agonist treatment, where regular check-ins with the prescriber during titration are important to foster discussions about expectations of the medication (e.g. potential effects, side-effects) [27–29]. Patients in the present study received a maximum daily dose of 120 mg (60 mg twice per day).

This follows from recent advances and conclusions drawn from the literature, for example, where a recent meta-analysis has revealed positive outcomes for patients receiving robust doses (defined as at least 60 mg/day) [13]. In studies where dextroamphetamine has been provided at lower fixed doses (e.g. maximum of either 30 or 60 mg/day) higher dropout rates and poorer outcomes have been reported [30] compared to in studies where higher doses have been offered (e.g. up to 110 mg/day) [31]. As known from the delivery of other pharmacological treatments such as oral methadone, while there are standardized thresholds for optimal dosing, positive outcomes are promoted when prescribers work with each patient to meet their personalized dosing needs [32].

Further to the medication dose, the ease with which patients could access dextroamphetamine also impacted on perceptions of the effectiveness of this medication. For example, some participants outlined the convenience of the on-site medication at the iOAT clinic. In prior studies, oral and injectable OAT clinics have been highlighted as suitable environments for the delivery of dextroamphetamine, whereby allied health professionals, including pharmacists, have a role to play in providing patients with medication reminders to promote adherence [10]. In this study however, the daily observed dispensation was seen to limit the potential effectiveness of the medication for many participants, by regimenting when the medication could be taken. For example, Dean reported wanting to take dextroamphetamine in line with his cravings, which did not always align with the time he visited the clinic for his iOAT dose. Similarly, William reported wanting to take the medication at different times each day depending on his work and sleep schedule. These findings suggest that where dextroamphetamine is delivered, flexibility around the timing of dose administration will be important to better engaging patients, particularly given the wide heterogeneity in substance use patterns, including different types of stimulant use, routes of administration, and frequencies of use [33].

This heterogeneity in stimulant use profiles provides further justification for considering diverse approaches to the delivery of dextroamphetamine in order to best respond to patients’ preferences. In this study, there was a preference among many participants for access to take-home (i.e. carries) doses. The provision of take-home doses of pharmacological treatments for substance use disorder have been greatly limited as a means of reducing possible medication diversion and promoting safety [34]. These restrictions directly affect the approach that can be taken with patients receiving dextroamphetamine in order to increase its uptake and effectiveness. In the context of COVID-19, there are increasing examples of the implementation of take-home doses being provided in the context of substance use disorder treatment to mitigate risks. If these measures are feasible and effective during COVID-19, implementation can continue post-COVID-19. For example, Switzerland offers up to seven days of take-home doses of iOAT [35], and take-home doses have also been provided in this study’s iOAT clinic to patients who are self-isolating [36]. Furthermore, in response to patient preferences, clinical protocols have been recently adjusted in the present study setting, allowing patients to take home their second dextroamphetamine dose of the day. Providing effective, patient-centered approaches means patient preferences must be acknowledged and incorporated into care [28, 37]. Take-home dosing can be implemented to respond to patients’ preferences and needs while also balancing potential concerns for safety, for example by scheduling regular visits, especially during medication initiation, to monitor for adverse events. In discussions about take-home dosing, concerns for medication diversion are often raised, however there has been relatively little consideration in the literature given to understanding factors (e.g. social, economic) that influence diversion [36].

The possible risks of diversion must be weighed against the challenges of more restrictive measures, which often come at the expense of patient autonomy, and which in some cases could deter patients from engaging at all with an otherwise potentially beneficial treatment. The illicit drug supply in British Columbia has become increasingly contaminated in recent years, and has contributed to record-high overdose mortality rates in the context of COVID-19. In this context, the possible elevated risk of harms for people relying on an illicit rather than prescribed stimulant supply must be also be considered in decisions about whether and how (i.e. take home or on-site) dextroamphetamine is prescribed. In the present study, and in the present moment of overlapping dual public health crises of overdose and COVID-19, there is an overwhelming patient preference for, and opportunity to offer and implement take-home dosing to meet patient needs and improve medication uptake and effectiveness.

There are a number of important limitations of the present study that must be considered in interpreting findings. First, this analysis was conducted with a very unique and specific sample of people with concurrent opioid and stimulant use disorder diagnoses, who were receiving treatment with dextroamphetamine and who had been regularly engaged in treatment at an iOAT clinic. Findings may not be generalizable to people with different demographic profiles receiving treatment in other settings. For example, participants had an average age of nearly 54 years, and may hold different substance use and treatment histories and treatment goals as compared to younger people who use illicit stimulants.

Furthermore, the concurrent opioid and stimulant use profile of participants is quite unique to this specific clinical setting, where patients are the only people in North America who have been receiving injectable diacetylmophine (pharmaceutical-grade heroin) or hydromorphone (an opioid analgesic) for the treatment of opioid use disorder for many years (nearly 10 years for some). These patients have shown significant reductions in illicit opioid use, and visit the clinic up to three times a day for their opioid medication [38]. This consistent daily (up to three times per day) engagement with the iOAT clinic persisted following access to dextroamphetamine, and there were no reported interactions between dextroamphetamine and the iOAT medication. Possible interactions of stimulant and opioid medications could be the focus of further pharmacologic studies, in particular among people who engage in daily illicit opioid use who might report on drug interactions which were not reported on in this study.

Second, the sample includes only a small number of women, which reflects the underrepresentation of women in the iOAT study setting [18]. As this medication expands to other settings, further work can be done to identify potential gender differences in preferences for the delivery of dextroamphetamine. Third, participants were eligible for inclusion in the study based on ever having attempted dextroamphetamine. As such, participants included in the study represent people who have had a wide range of exposure to the medication which could impact on the diversity of overall experiences and perceptions of effectiveness. Fourth, while many clinical trials have investigated the effectiveness of this medication separately among people with cocaine or methamphetamine use disorder, participants in the present study reported cocaine and/or methamphetamine use. Nevertheless, a prior qualitative study investigating patients’ illicit stimulant use in this study setting (iOAT clinic) found no differences across themes by reported illicit stimulant use type [17]. Furthermore, the present study includes only participants who remained engaged with the iOAT clinic and thus the perspective of people who received dextroamphetamine but were not well engaged in iOAT are not represented. People who were hesitant or resistant to attempting dextroamphetamine were not included in the study sample. This presents a sample that could be the focus of future research to help inform modifications to the delivery of dextroamphetamine prescribing to engage a broader range of patients in treatment.

It is also important to highlight that this study took place both prior to and following the declaration of COVID-19 as a public health emergency in British Columbia. This meant that data were collected both in person, at a confidential research office, and by telephone. We acknowledge that the strengths of qualitative data collection rest heavily on both the context and setting of the interview. In our experience, in person data collection has yielded rich insights and discussion. Nevertheless, we were able to rely on pre-existing rapport with participants when conducting telephone interviews, to maintain rich discussion. Furthermore, prior studies have found that telephone interviews may allow respondents to feel more comfortable and relaxed, and that there is no evidence to suggest that telephone interviews produce lower quality data [39].

This qualitative study relied on participants’ accounts of their experiences with dextroamphetamine, and self-reported patterns of illicit stimulant use. Prior studies have concluded the reliability of participants’ accounts of their substance use where rapport exists, and where the person inquiring has no power or control over their treatment or care [40]. In the present study, the interviewer held existing rapport with participants, having regularly administered self-reported questionnaires about their illicit substance use patterns for related studies in prior years. Furthermore, given previous regular contact for existing research, the boundary between research team and the clinical team was already well established and understood by participants.

Lastly, this study cannot confirm or deny the efficacy of dextroamphetamine for the treatment of stimulant use disorder. Instead, this study offers an exploration of patient perceptions of dextroamphetamine, which cannot be captured by other study designs (e.g. clinical trials) where quantitative outcome assessments are made to determine effectiveness.

## Conclusion

In the context of continued investigation of pharmacological treatments for stimulant use disorder, the present study has highlighted how the study of clinical outcomes could be extended to account for factors that contribute to perceptions of effectiveness from the perspective of patients. This could include a movement beyond a focus on the pharmacological medication itself (e.g. which medications are the “most” effective) to considering how the delivery of each of these medications could be adapted to better meet patients’ needs and preferences. Patients in both clinical trial and community treatment settings could benefit when researchers and clinicians consider broadening the operationalization of what makes an “effective” treatment. In practice, elements of treatment delivery (e.g. dosing and dispensation protocols) can be adjusted to allow for various scenarios (e.g. on site vs. take home dosing) by which dextroamphetamine and other pharmacological stimulants could be implemented to provide “effective” treatment for people with a wide range of treatment goals and needs.

## Data Availability

All data is in the form of transcripts from interviews and focus groups. Data sharing is not applicable to this article as no datasets were generated or analysed during the current study.

## Abbreviations

ADHD: Attention decific hyperactivity disorder
OAT: opioid agonist treatment
iOAT: injectable opioid agonist treatment
RUTH: Research on the Utilization of Therpeutic Hydromorphone
